# *Cordyceps militaris* Enhances Wound Repair Through Regulation of HIF-1α, TGF-β1, and SIRT1/Nrf2/HO-1 Signaling in Diabetic Skin

**DOI:** 10.3390/life16010117

**Published:** 2026-01-13

**Authors:** Tzu-Kai Lin, Chia-Lun Tsai, Bruce Chi-Kang Tsai, Chia-Hua Kuo, Tsung-Jung Ho, Dennis Jine-Yuan Hsieh, Wei-Wen Kuo, Chih-Yang Huang, Pei-Ying Lee

**Affiliations:** 1 Department of Dermatology, Hualien Tzu Chi Hospital, Buddhist Tzu Chi Medical Foundation, Hualien 970473, Taiwan; 2 Cardiovascular and Mitochondrial Related Disease Research Center, Hualien Tzu Chi Hospital, Buddhist Tzu Chi Medical Foundation, Hualien 970473, Taiwan; 3 Laboratory of Exercise Biochemistry, The Education University of Hong Kong, New Territories, Hong Kong; 4 School of Physical Education and Sports Science, Soochow University, Suzhou 215006, China; 5 Department of Movement Sciences and Sports Training, School of Sport Sciences, University of Jordan, Amman 11942, Jordan; 6 Department of Chinese Medicine, Hualien Tzu Chi Hospital, Buddhist Tzu Chi Medical Foundation, Hualien 970473, Taiwan; 7 Integration Center of Traditional Chinese and Modern Medicine, Hualien Tzu Chi Hospital, Buddhist Tzu Chi Medical Foundation, Hualien 970473, Taiwan; 8 Department of Medical Laboratory and Biotechnology, Chung Shan Medical University, Taichung 402306, Taiwan; 9 Clinical Laboratory, Chung Shan Medical University Hospital, Taichung 402306, Taiwan; 10 Department of Biological Science and Technology, College of Life Sciences, China Medical University, Taichung 406040, Taiwan; 11 Ph.D. Program for Biotechnology Industry, China Medical University, Taichung 406040, Taiwan; 12 School of Pharmacy, China Medical University, Taichung 406040, Taiwan; 13 Graduate Institute of Biomedical Sciences, China Medical University, Taichung 404333, Taiwan; 14 Department of Medical Research, China Medical University Hospital, China Medical University, Taichung 404327, Taiwan; 15 Department of Medical Laboratory Science and Biotechnology, Asia University, Taichung 413305, Taiwan; 16 Division of Basic Medical Sciences, College of Nursing, Tzu Chi University, Hualien 970374, Taiwan

**Keywords:** *Cordyceps militaris*, wound healing, diabetes, skin care

## Abstract

Chronic diabetic wounds are characterized by persistent inflammation, impaired angiogenesis, oxidative stress, and defective tissue remodeling, leading to delayed healing. *Cordyceps militaris*, a medicinal fungus with known anti-inflammatory and antioxidant properties, has shown therapeutic potential in metabolic disorders; however, its role in diabetic wound repair remains unclear. In this study, we evaluated the wound-healing effects of an aqueous extract of *C. militaris* using in vitro keratinocyte models and a streptozotocin-induced diabetic mouse model. *C. militaris* treatment significantly accelerated wound closure, improved epidermal regeneration, and enhanced skin barrier integrity. Mechanistically, *C. militaris* restored HIF-1α and TGF-β1 expression, promoted cell proliferation and fibroblast activation, and increased the expression of matrix metalloproteinases MMP-1 and MMP-2, indicating enhanced extracellular matrix remodeling. In parallel, excessive inflammatory responses were attenuated, as evidenced by reduced IL-6 and TNF-α levels, along with activation of SIRT1/Nrf2/HO-1 antioxidant signaling pathways. Collectively, these findings demonstrate that *C. militaris* promotes a balanced wound-healing microenvironment and represents a promising natural therapeutic candidate for the treatment of diabetic wounds.

## 1. Introduction

Diabetes mellitus (DM) is a prevalent metabolic disorder that significantly impacts millions of individuals across the globe. The global prevalence of diabetes has been increasing at an alarming rate, with current estimates indicating over 500 million affected individuals. If effective preventive measures and therapeutic interventions are not implemented, projections suggest that the number of cases could surpass 700 million within the next two decades, according to the report of the International Diabetes Federation in 2021 [1]. As a result, DM poses a considerable burden on public health systems and individuals alike. This chronic condition arises due to impairments in insulin function, leading to sustained hyperglycemia over an extended period. Given the profound implications of diabetes on morbidity, mortality, and healthcare expenditures, urgent efforts are required to develop and implement comprehensive strategies aimed at mitigating its growing impact [2,3]. DM is associated with a wide range of chronic complications that adversely affect multiple organ systems in affected individuals. Prolonged hyperglycemia can lead to significant damage to the nervous system, eyes, cardiovascular system, and kidneys, contributing to debilitating conditions such as diabetic neuropathy, retinopathy, cardiovascular diseases, and nephropathy [4,5]. DM is also associated with a variety of dermatological complications. Previous studies have demonstrated that several structural and functional characteristics of the skin undergo significant alterations in individuals with DM. Notably, changes are observed in skin hydration levels, filaggrin expression, epidermal thickness, epidermal differentiation, and keratinocyte proliferation. Additionally, impairments in immune function and skin barrier integrity are evident, as reflected by the decreased levels of T-cell activity, collagens, antimicrobial peptides, stratum corneum lipids, lamellar bodies, and their associated enzymes [6]. Furthermore, an upregulation of matrix metalloproteinase activity has been reported [7]. The inflammatory infiltration is exacerbated [8]. Skin properties in diabetic skin are deteriorated [9]. These alterations increase the susceptibility of diabetic individuals to skin infections. Among the most severe cutaneous complications are chronic ulcers, which result from impaired wound-healing processes characteristic of diabetic skin. Due to the high risk of secondary infections, these ulcers frequently progress to severe tissue damage, ultimately necessitating limb amputation in affected patients [6]. Hence, the development of effective therapeutic strategies to enhance wound healing in diabetic patients is essential.

With demonstrated protective properties against various stresses and diseases, Chinese herbal medicines emerge as promising candidates for the development of innovative therapeutic approaches focused on promoting and maintaining skin health [10,11,12,13,14]. *Cordyceps militaris* (*C. militaris*), a member of the *Cordyceps genus*, is an entomopathogenic fungus that has attracted considerable scientific interest in recent decades. This organism is distinguished by its complex chemical profile and a wide range of bioactive compounds, which have been extensively studied for their therapeutic potential. The pharmacological significance of *C. militaris* has been increasingly recognized, with research highlighting its multifaceted biological activities and potential applications in medicine [15,16,17,18]. For DM treatment, previous studies present some benefits related to *C. militaris* and DM. *C. militaris* improves beneficial gut microbiota growth and modulates intestinal flora structure, thereby enhancing metabolic pathways associated with DM [19]. Similarly, polysaccharide fractions from *C. militaris* also modulate gut microbiota by increasing beneficial bacteria and reducing harmful taxa, thereby inhibiting the colonic TLR4/NF-κB pathway and enhancing intestinal barrier integrity. These changes improved glucose metabolism, serum lipids, and hormone secretion [20]. Additionally, Cordycepin from *C. militaris* reduces oxidative stress, inflammation, and apoptosis in high glucose-induced HK-2 cells by modulating the miR-193b-5p/MCL-1 pathway. In a mouse model, it improves renal function and pathology, further supporting its nephroprotective role [21]. Cordycepin alleviates oxidative stress, restores the blood-testis barrier, and improves spermatogenesis by activating the SIRT1/Foxo3a pathway. This activation upregulates antioxidant enzymes, reducing apoptosis and oxidative damage against diabetes-induced testicular damage [22]. These findings support the potential of *C. militaris* as a functional food for the management of DM and its complications.

Although *C. militaris* offers numerous health benefits for DM and has shown potential in managing DM and its complications, limited research has explored its protective effects on diabetic skin. Therefore, the primary objective of this study was to investigate the ability of *C. militaris* to enhance wound healing in diabetic skin using both in vitro and in vivo models. Through this research, we aimed to provide a deeper understanding of its therapeutic potential in promoting skin regeneration and improving diabetic wound healing.

## 2. Materials and Methods

### 2.1. Study Period

All in vitro and in vivo experiments were conducted at Hualien Tzu Chi Hospital and the Laboratory Animal Center of Tzu Chi University, Taiwan, between 1 April 2022 and 31 July 2024.

### 2.2. Preparation of C. militaris Aqueous Extraction

A fine powder was obtained by grinding a total of 26 g of dried *C. militaris*, cultivated and provided by the Buddhist Tzu Chi Medical Foundation, using a mechanical grinder. This powder was then homogenized with 250 mL of distilled water. To optimize the extraction efficiency, the suspension was subjected to ultrasonic agitation (frequency: 40 KHz, output power: 900 W) at a maintained temperature of 25 °C for a duration of 1.5 h in DELTA^®^ Ultrasonic cleaner (DC900H, DELTA Ultrasonic Co., Ltd., New Taipei, Taiwan). Following this, the mixture was refrigerated at 4 °C for over 12 h to facilitate the thorough extraction of bioactive compounds. The resulting crude extract was centrifuged at 4000× *g* for 20 min at 4 °C to separate and discard any insoluble residues. The supernatant was subsequently filtered twice through qualitative filter paper (pore size: 6 μm, No. 80215102, ADVANTEC, TOYO ROSHI KAISHA, Ltd., Tokyo, Japan). The filtrate was sterilized by passage through a 0.45 µm membrane filter, eliminating any residual particulates or microbial contaminants. The final concentration of *C. militaris* aqueous extract was quantified as 47 mg/mL. The samples were submitted to Protech Technology Enterprise Co., Ltd. (Taipei, Taiwan) for high-performance liquid chromatography (HPLC) and mass spectrometry (MS) analysis to determine the levels of cordycepin and adenosine. The analyses were carried out using an Agilent 1260 HPLC system equipped with a Phenomenex Kinetex Phenyl-Hexyl-100A column (100 mm × 2.1 mm, 2.6 μm, Torrance, CA, USA) in conjunction with an AB Sciex QTRAP 5500 mass spectrometer (SCIEX, Framingham, MA, USA).

### 2.3. Cell Culture

HaCaT keratinocytes (ABC-TC536S) were procured from Accegen Biotechnology (Fairfield, NJ, USA). The cells were cultured in Gibco™ Dulbecco’s Modified Eagle Medium (12100038, Thermo Fisher Scientific, Waltham, MA, USA) supplemented with 10% (*v*/*v*) fetal bovine serum (89510186, Avantor, Radnor, PA, USA) and 1% (*v*/*v*) Gibco™ penicillin–streptomycin (15140122, Thermo Fisher Scientific). They were maintained under standard cell culture conditions in a humidified incubator at 37 °C with 5% CO_2_. To ensure cell integrity, routine mycoplasma contamination screening was conducted using the TOOLS Mycoplasma Detection Kit (TTB-GBC8, BIOTOOLS, New Taipei City, Taiwan). For experimental treatments, a high-glucose medium containing 55 mM glucose was utilized.

### 2.4. Wound-Healing Assay

A total of 1 × 10^5^ cells were plated in a 12-well culture plate and maintained under standard culture conditions until they attained 80% confluence. Upon reaching the desired confluence, a linear scratch was introduced into the cell monolayer using a 1 mL pipette tip to mimic a wound. The cells were then gently rinsed with phosphate-buffered saline (PBS) to eliminate any detached cells, followed by the replacement of the medium with fresh culture medium. Subsequently, the cells were exposed to *C. militaris* aqueous extract at concentrations of 5 and 30 µg/mL and incubated for 24 h to evaluate the effect of *C. militaris* aqueous extract on wound closure and cell migration [23,24,25]. Following the incubation period, the samples were washed three times with PBS to remove any residual treatment compounds and cellular debris. The progression of wound healing and cell migration was then observed and recorded using an Olympus IX71 microscope (Olympus Corporation, Tokyo, Japan).

### 2.5. In Vitro Immunofluorescence Assay

A total of 1 × 10^5^ cells were cultured in 12-well chamber slides and incubated for 24 h. Subsequently, the cells were treated with varying concentrations of *C. militaris* aqueous extract and glucose, followed by an additional 24-h incubation period [23,24,25]. After treatment, the cells were washed with PBS, fixed with 4% paraformaldehyde at 25 °C for 30 min, and permeabilized using 0.1% Triton X-100 at 4 °C for 30 min. To prevent nonspecific binding, the samples were blocked with 1% horse serum in PBS at 25 °C for 60 min. Primary antibodies targeting HIF-1α (1:50, sc-13515, Santa Cruz Biotechnology, Santa Cruz, CA, USA) and TGFβ1 (1:50, sc-130348, Santa Cruz Biotechnology) were then applied, and the cells were incubated at 4 °C for 12 h. Following PBS washes, the samples were incubated with either Goat anti-Mouse IgG (H + L) Cross-Adsorbed Secondary Antibody (Alexa Fluor™ 488, A11001, Invitrogen, Carlsbad, CA, USA) or Goat anti-Mouse IgG (H+L) Highly Cross-Adsorbed Secondary Antibody (Alexa Fluor™ 594, A11032, Invitrogen) at 25 °C for 60 min. After additional PBS washes, the nuclei were counterstained with DAPI (D9542, Sigma-Aldrich, St. Louis, MO, USA) for 10 min. The samples were then subjected to three final PBS washes before fluorescence imaging was performed using an OLYMPUS BX53 fluorescence microscope (Olympus Corporation, Shinjuku-ku, Tokyo, Japan) [26].

### 2.6. Animal Experiment

All animal experiments were approved by the Institutional Animal Care and Ethics Committee of Hualien Tzu Chi Hospital (Hualien, Taiwan) (Animal approval No.: 111-11) and were conducted in accordance with the Guide for the Care and Use of Laboratory Animals issued by the National Institutes of Health (NIH). Ten-week-old, healthy, male, hairless SKH2/J mice (strain 002335, originally from The Jackson Laboratory, Bar Harbor, ME, USA) were bred and provided by the Laboratory Animal Centre at Tzu Chi University (Hualien, Taiwan). A total of 32 mice were randomly divided into four groups: normal group, normal +*C. militaris* group, DM group, and DM +*C. militaris* group. Each group consisted of eight mice. The animals were housed in a controlled environment, maintained at a temperature of 22 ± 2 °C and a relative humidity of 50 ± 5%, under a 12 h light/dark cycle. All mice were provided with standard laboratory chow (Lab Diet 5001; PMI Nutrition International Inc., Brentwood, MO, USA) and had unrestricted access to tap water.

Type 1 diabetes was induced by intraperitoneal injection of streptozotocin (STZ, sc-200719, Santa Cruz Biotechnology) at a dose of 100 mg/kg body weight, prepared in citrate buffer (pH 4.5). Blood glucose levels were monitored using Accu-Chek^®^ Guide test strips and an Accu-Chek^®^ Guide meter (Fritz Hoffmann-La Roche AG, Basel, Switzerland) [27]. Mice were classified as diabetic if their blood glucose levels remained above 300 mg/dL for 48 h post-STZ administration.

All animal procedures, including anesthesia and euthanasia, were performed in accordance with the guidelines of the American Veterinary Medical Association. Before wound induction, the dorsal skin of each mouse was disinfected with 75% ethanol and rinsed with sterile saline. Under isoflurane anesthesia (3% for induction and 1–2% for maintenance in oxygen), two full-thickness excisional wounds (6 mm in diameter) were created on either side of the dorsal midline using a sterile biopsy punch. The wound area was measured daily using a standard ruler. In the *Cordyceps militaris*-treated group, wounds were topically administered with 10 μL of *C. militaris* aqueous extract (10 mg/mL) once daily for 14 consecutive days, whereas the control group received an equivalent volume of vehicle. At the end of the experimental period (day 14), mice were humanely euthanized by excessive carbon dioxide inhalation, and skin tissues from the wound area were collected for further analysis.

### 2.7. In Vivo Skin Examination: Trans-Epidermal Water Loss (TEWL)

The assessment of trans-epidermal water loss (TEWL) was conducted on days 7 and 10 of *Cordyceps militaris* treatment to evaluate skin barrier integrity. TEWL measurements were performed using the Tewameter TM300 probe, which was integrated into the Multi Probe Adapter (MPA) system (Courage + Khazaka GmbH, Cologne, Germany). The procedure took place in a controlled, air-conditioned environment to minimize external variability. The room conditions were maintained with a stable temperature of 22 ± 2 °C and a relative humidity of 50 ± 5% to improve data reliability by reducing potential fluctuations due to environmental factors [28].

### 2.8. Frozen Section

The skin tissue was initially fixed in 2% paraformaldehyde for 20 s, followed by sequential incubation in sucrose solutions and subsequent embedding in O.C.T. embedding compound. The embedded tissue was then cryosectioned into 10 μm thick slices using the Leica CM1950 cryostat (Leica Biosystems, Deer Park, IL, USA) to ensure precise sectioning. The obtained tissue sections were carefully mounted onto glass microscope slides for subsequent histological analysis.

### 2.9. Hematoxylin and Eosin (H&E) Staining

Cryostat tissue sections were first fixed at 65 °C and subsequently rinsed with double-distilled water (ddH_2_O) to remove the O.C.T. embedding compound. The sections were then incubated with hematoxylin solution (GHS316-500ML, Sigma-Aldrich) for 1 min, followed by washing with ddH_2_O. Eosin Y solution (HT110116, Merck, Darmstadt, Germany) was subsequently applied for 30 s and rinsed with 95% ethanol. After air-drying, the slides were mounted using a mounting medium (M1289, Sigma-Aldrich). Histological images were captured using an OLYMPUS BX53 fluorescence microscope.

### 2.10. Tissue Immunofluorescence Staining

To begin, cryostat sections were fixed in 10% neutral buffered formalin for 15 min at 25 °C. Following fixation, the sections underwent three sequential washes with phosphate-buffered saline (PBS), each lasting 5 min at 25 °C. Tissue permeabilization was achieved by incubating the sections in 0.1% Triton X-100 for 15 min at 25 °C, followed by an additional three washes with PBS under identical conditions. To minimize non-specific antibody binding, a blocking step was performed using 1% bovine serum albumin (BSA) for one hour in a humidified chamber at 25 °C. Subsequently, the sections were exposed to the primary antibody, prepared at a 1:100 dilution in 1% BSA. The primary antibodies utilized in this study included anti-IL-6 (1:50, sc-130326, Santa Cruz Biotechnology), anti-MMP-1 (1:50, sc-21731, Santa Cruz Biotechnology), anti-MMP-2 (1:50, sc-13595, Santa Cruz Biotechnology), anti-TGF-β1 (1:50, sc-130348, Santa Cruz Biotechnology), anti-HIF-1α (1:50, sc-13515, Santa Cruz Biotechnology), anti-Vimentin (1:50, sc-32322, Santa Cruz Biotechnology), anti-PCNA (1:50, sc-56, Santa Cruz Biotechnology), anti-TNF-α (1:50, sc-52746, Santa Cruz Biotechnology), anti-SIRT1 (1:50, sc-74465, Santa Cruz Biotechnology), anti-Nrf2 (1:100, ab89443, Abcam, Cambridge, UK), and anti-HO-1 (1:50, sc-136961, Santa Cruz Biotechnology). These antibodies were applied to the sections, followed by incubation at 4 °C for 12 h. Post-incubation, excess primary antibody was removed through three washes with PBS, each lasting 5 min at 25 °C.

Next, sections were treated with an appropriate fluorescently labeled secondary antibody, diluted at 1:1000 in 1% BSA, and incubated for one hour at 25 °C in the dark to preserve fluorescence integrity. Three additional PBS washes, each lasting 5 min at 25 °C, were then performed under the same light-protected conditions. Finally, the stained sections were mounted using a DAPI-containing mounting medium (D9542, Sigma-Aldrich) and secured with a coverslip. Imaging was conducted using an OLYMPUS BX53 fluorescence microscope.

### 2.11. Statistical Analysis

Data obtained from at least three independent experiments are presented as the mean ± standard deviation (SD). Statistical comparisons between groups were performed using Student’s *t*-test. A *p*-value of less than 0.05 was regarded as indicative of statistical significance.

## 3. Results

### 3.1. Bioactive Compounds of the C. militaris Aqueous Extract via HPLC/MS Analysis

HPLC/MS analysis was conducted to determine the presence and quantify the levels of the major bioactive constituents in the *C. militaris* aqueous extract. Particular attention was given to adenosine and cordycepin, which are widely recognized as the primary pharmacologically active compounds of *C. militaris* and are closely associated with its biological and therapeutic activities. The analysis revealed that both compounds were readily detectable in the extract. As summarized in Table 1 and the Supplementary Materials, the concentration of adenosine in the aqueous extract was 84.60 μg/mL, while the concentration of cordycepin was 51.30 μg/mL. These quantitative results indicate that the aqueous extract provides a considerable amount of both compounds.

### 3.2. C. militaris Treatment Enhances Wound-Healing Ability In Vitro

**Figure 1:**
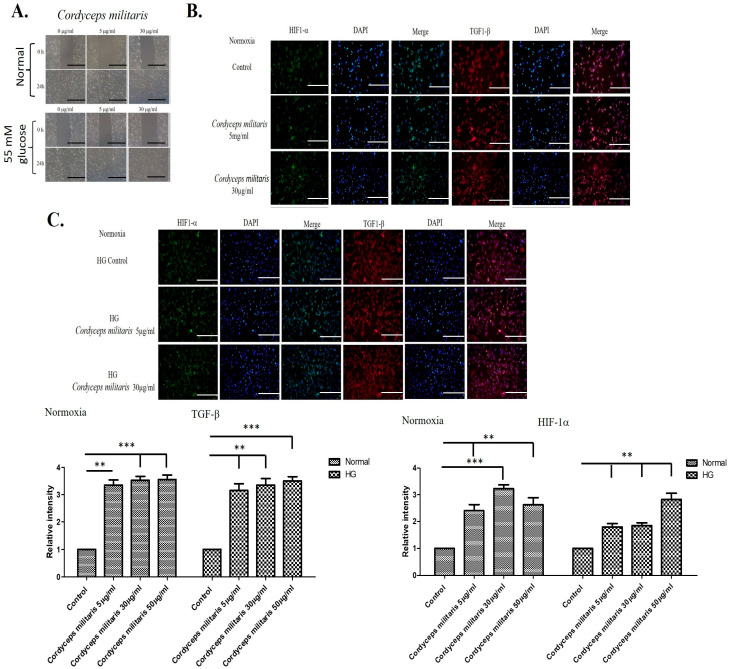
*C. militaris* enhances wound healing by promoting cell migration and upregulating key regulatory factors: (**A**) In vitro wound-healing assay showing increased cell migration following treatment with *C. militaris* at 5 µg/mL and 30 µg/mL under normal and high-glucose conditions. The scale bar was 200 μm. (**B**,**C**) Immunofluorescence staining indicating upregulated expression of HIF-1α and TGF-β1 in cells treated with *C. militaris*, suggesting its role in activating key pathways involved in wound repair in both normal and high glucose conditions. The scale bar is 100 μm. ** *p* < 0.01and *** *p* < 0.001.

To evaluate the potential wound-healing properties of *C. militaris*, an in vitro wound-healing assay was performed. The results demonstrated that treatment with *C. militaris* at concentrations of 5 µg/mL and 30 µg/mL obviously enhanced cell migration and differentiation under both normal and high-glucose conditions (Figure 1A). These findings suggest that *C. militaris* may contribute to improved wound repair processes by facilitating cellular movement and regenerative activity. Furthermore, given the crucial roles of HIF-1α and TGF-β1 in the regulation of wound healing [29,30], their expression levels were assessed using immunofluorescence staining. The staining results indicated that *C. militaris* treatment led to an upregulation of both HIF-1α and TGF-β1 expression in cells cultured under normal and high-glucose conditions (Figure 1B,C). This suggests that the enhanced wound-healing effects of *C. militaris* may be mediated by the activation of these key regulatory factors.

### 3.3. A Hairless Mouse Model Demonstrates the Efficacy of C. militaris Treatment in Enhancing Wound Healing Through the Regulation of Multiple Factors

**Figure 2:**
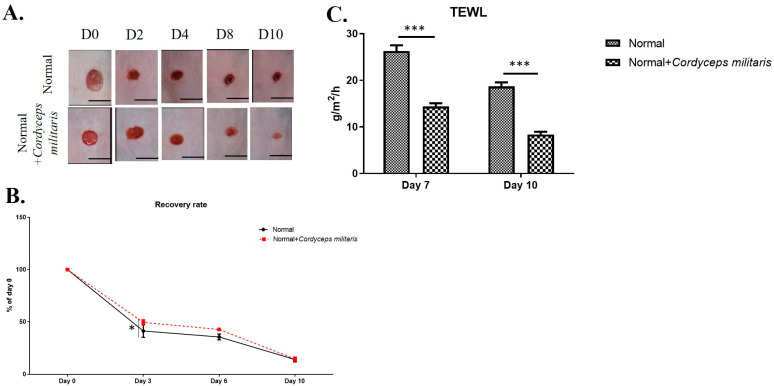
*C. militaris* accelerates wound healing and enhances skin barrier function in a normal hairless mouse model: (**A**,**B**) Representative images and quantification of wound closure showing significantly faster healing in the *C. militaris*-treated group compared to the control group. The scale bar was 3 mm. (**C**) Measurement of trans-epidermal water loss (TEWL) indicating improved skin barrier restoration in the *C. militaris*-treated group, suggesting enhanced skin integrity and reduced risk of secondary infection. n = 8; * *p* < 0.05; *** *p* < 0.001.

**Figure 3:**
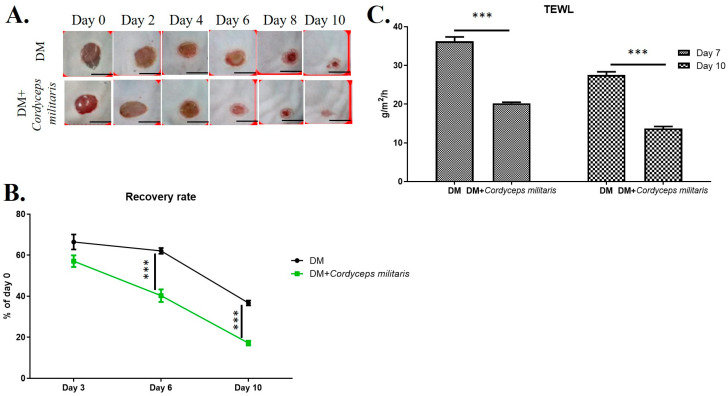
*C. militaris* accelerates wound healing and enhances skin barrier function in a diabetic hairless mouse model: (**A**,**B**) Representative images and quantification of wound closure showing significantly faster healing in the *C. militaris*-treated group compared to the control group. The scale bar was 3 mm. (**C**) Measurement of trans-epidermal water loss (TEWL) indicating improved skin barrier restoration in the *C. militaris*-treated group, suggesting enhanced skin integrity and reduced risk of secondary infection. n = 8; *** *p* < 0.001.

**Figure 4:**
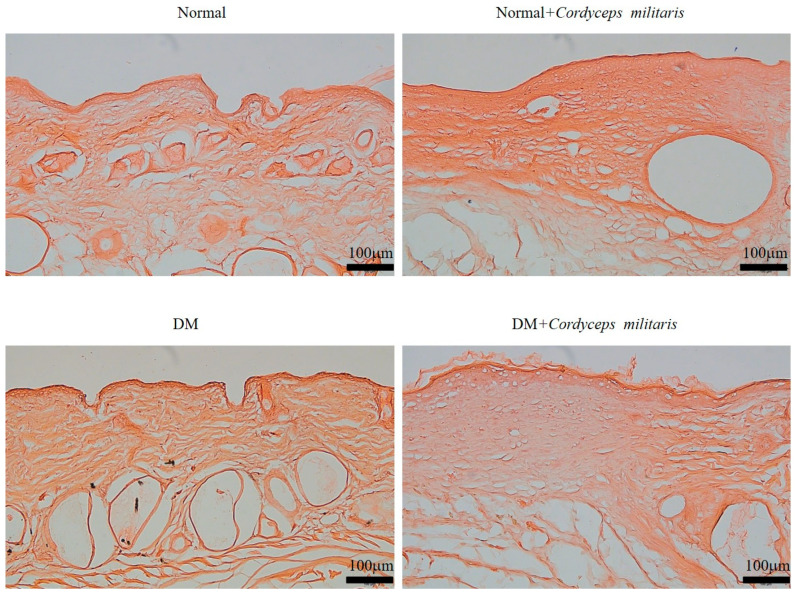
*Cordyceps militaris* extract improves skin architecture in diabetic wounds. Hematoxylin and eosin (H&E)-stained sections showing skin morphology in the indicated experimental groups. The diabetes mellitus (DM) group exhibited a markedly reduced skin thickness compared with the non-diabetic and treatment groups. Administration of *C. militaris* extract substantially restored skin thickness and improved overall tissue organization, indicating a therapeutic effect on diabetic wound healing. Scale bar: 100 µm.

Wound healing is a complex and highly regulated biological process involving various cellular and molecular mechanisms. To investigate the potential therapeutic benefits of *C. militaris* in promoting wound healing, a hairless mouse model was employed. In both the normal group and the DM group, during the wound-healing period, we observed a marked enhancement in wound closure in the *C. militaris*-treated groups compared to the untreated group. The wounds in the treatment group exhibited faster healing rates and a smaller wound area throughout the observation period, thereby reducing the risk of secondary wound infection (Figure 2A,B and Figure 3A,B). Additionally, to further evaluate the effect of *C. militaris* on skin integrity and barrier function, TEWL was measured. The results indicated a significant reduction in water loss in the *C. militaris*-treated group compared to the control, demonstrating improved skin barrier restoration (Figure 2C and Figure 3C). Histological morphology was evaluated by H&E staining. The results demonstrated that the skin layer in the DM group was markedly thinner than that in the other groups (Figure 4). In contrast, treatment with *C. militaris* extract significantly improved skin thickness, suggesting its potential therapeutic effect.

**Figure 5:**
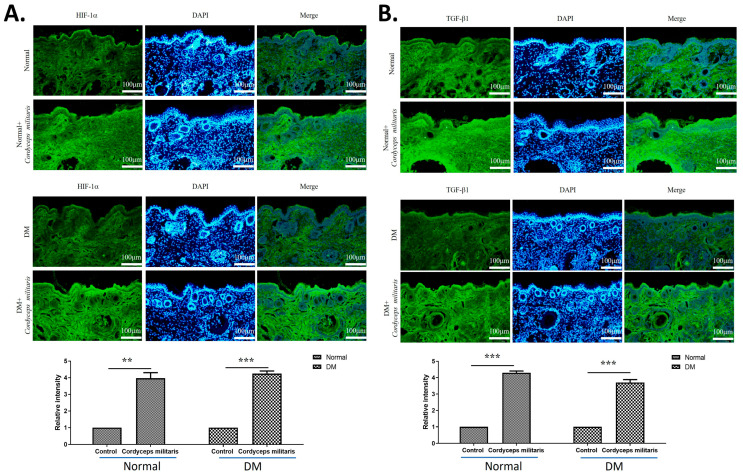
*Cordyceps militaris* enhances HIF-1α and TGF-β1 expression during wound healing. Immunofluorescence images showing the expression of (**A**) hypoxia-inducible factor-1α (HIF-1α) and (**B**) transforming growth factor-β1 (TGF-β1) in wound tissues from the indicated experimental groups. Compared with the DM group, *C. militaris* treatment markedly increased HIF-1α and TGF-β1 immunoreactivity, indicating activation of pro-repair pathways. These findings are consistent with previous in vitro studies demonstrating the ability of *C. militaris* to modulate hypoxic responses and promote tissue regeneration. Scale bar: 100 µm. ** *p* < 0.01 and *** *p* < 0.001.

**Figure 6:**
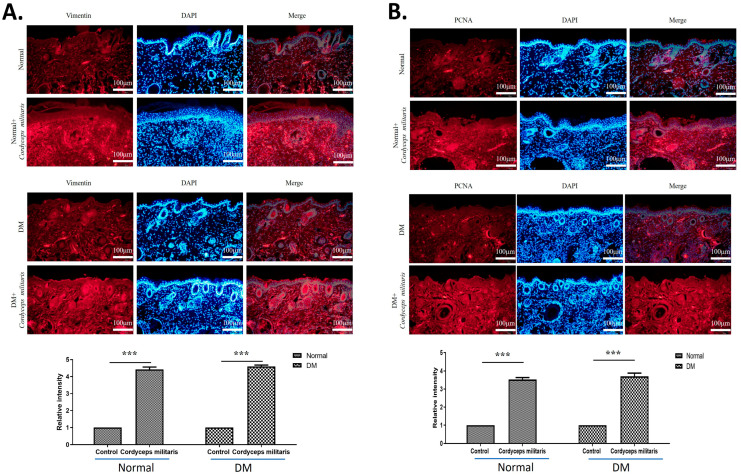
*Cordyceps militaris* promotes fibroblast activation, cell proliferation, and epidermal maturation in wound tissue. Immunofluorescence images showing the expression of (**A**) vimentin and (**B**) proliferating cell nuclear antigen (PCNA) in wound sections from the indicated experimental groups. *C. militaris* treatment markedly increased vimentin-positive cells, indicating enhanced fibroblast migration and epithelial–mesenchymal transition–related processes, as well as elevated PCNA expression, reflecting increased cellular proliferation. These changes are consistent with accelerated tissue regeneration during wound healing. Scale bar: 100 µm. *** *p* < 0.001.

**Figure 7:**
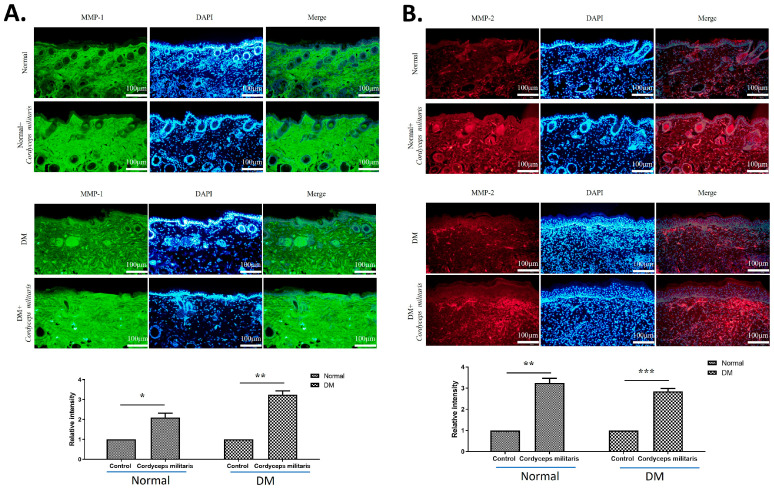
*Cordyceps militaris* enhances extracellular matrix remodeling during wound healing. Immunofluorescence images showing the expression of (**A**) matrix metalloproteinase-1 (MMP-1) and (**B**) matrix metalloproteinase-2 (MMP-2) in wound tissues from the indicated experimental groups. Compared with the DM group, *C. militaris* treatment markedly increased MMP-1 and MMP-2 expression, indicating enhanced extracellular matrix remodeling. The upregulation of these proteases supports facilitated migration of keratinocytes, fibroblasts, and endothelial cells into the wound bed, thereby promoting tissue repair and re-epithelialization. Scale bar: 100 µm. * *p* < 0.05, ** *p* < 0.01, and *** *p* < 0.001.

Further, to elucidate the molecular mechanisms underlying the wound-healing properties of *C. militaris*, immunofluorescence staining was performed to assess the expression of key regulatory factors. The results revealed a notable upregulation of HIF-1α (Figure 5A) and TGF-β1 (Figure 5B) in the *C. militaris*-treated group. These observations are consistent with previous in vitro studies demonstrating the role of *C. militaris* in modulating cellular responses to hypoxia and promoting tissue repair. Moreover, the expression of vimentin (Figure 6A) and proliferating cell nuclear antigen (PCNA) (Figure 6B)—all critical regulators of wound healing—was obviously increased following *C. militaris* treatment. Vimentin is known for its role in epithelial–mesenchymal transition and fibroblast migration, PCNA is a well-established marker of cell proliferation, and loricrin is a key component of the epidermal differentiation complex involved in the formation of the skin barrier [31,32,33]. The expression of MMP-1 (Figure 7A) and MMP-2 (Figure 7B) was also notably increased following *C. militaris* treatment. MMP-1 and MMP-2 play critical roles in wound healing by regulating extracellular matrix remodeling. These enzymes facilitate cell migration by degrading collagen and other matrix components, thereby allowing keratinocytes, fibroblasts, and endothelial cells to migrate into the wound site and promote tissue repair and re-epithelialization [34,35,36].

**Figure 8:**
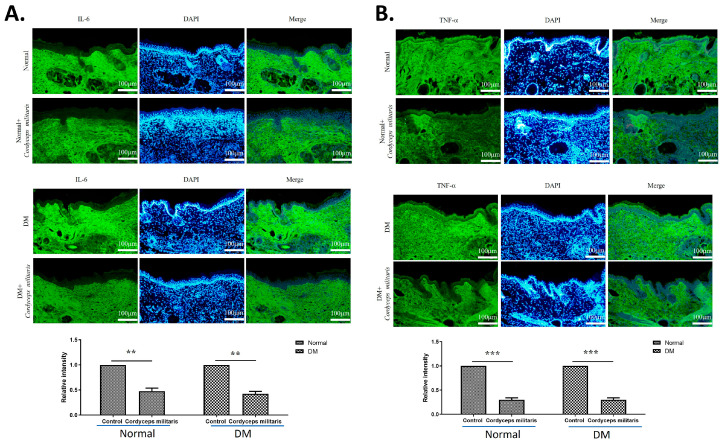
*Cordyceps militaris* attenuates pro-inflammatory cytokine expression in wound tissue. Immunofluorescence images showing the expression of the pro-inflammatory cytokines (**A**) interleukin-6 (IL-6) and (**B**) tumor necrosis factor-α (TNF-α) in wound sections from the indicated experimental groups. Compared with the DM group, *C. militaris* treatment markedly reduced IL-6 and TNF-α immunoreactivity, indicating suppression of excessive inflammation. This anti-inflammatory effect may contribute to the improved wound-healing outcome observed following *C. militaris* administration. Scale bar: 100 µm. ** *p* < 0.01, and *** *p* < 0.001.

In addition to promoting factors associated with wound repair, *C. militaris* treatment led to a notable decrease in IL-6 and TNF-α expression (Figure 8). IL-6 is a key mediator of the early inflammatory response and is essential for proper progression of the wound-healing process. During the remodeling phase, IL-6 levels normally decline and return to baseline. However, sustained or excessive IL-6 expression may impair tissue repair and is often associated with delayed or dysfunctional wound healing [37]. TNF-α is a pro-inflammatory cytokine that plays a pivotal role in chronic inflammation and impaired cutaneous wound healing [38,39]. The suppression of these inflammatory cytokines suggests that *C. militaris* exerts anti-inflammatory effects, which may contribute to a more favorable wound-healing environment by reducing excessive inflammation and promoting tissue regeneration. The findings indicate the therapeutic potential of *C. militaris* as a natural agent for enhancing wound healing. By accelerating wound closure, improving skin barrier function, modulating key regulatory factors, and exerting anti-inflammatory effects, *C. militaris* demonstrates a multifaceted approach to wound repair in normal and DM mice.

### 3.4. Activation of the SIRT1/Nrf2 Signaling Pathway by Cordyceps militaris Modulates Wound Healing

**Figure 9:**
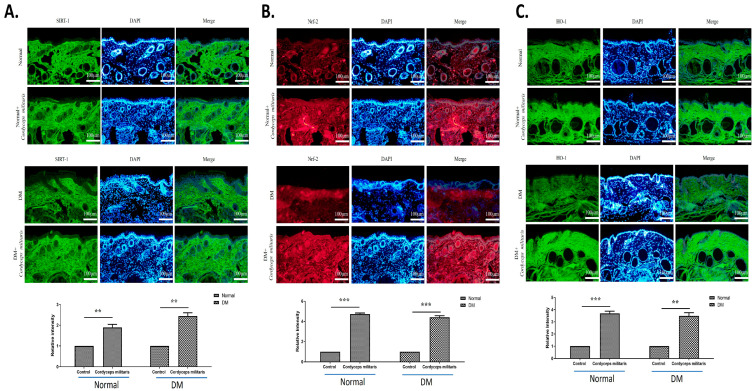
*Cordyceps militaris* enhances wound healing through activation of the SIRT1/Nrf2 signaling pathway: (**A**,**B**) Immunofluorescence staining showing upregulated expression of SIRT1 and Nrf2 in the *C. militaris*-treated group, indicating activation of protective cellular pathways. (**C**) Increased expression of HO-1 in the *C. militaris*-treated group, a downstream effector of the SIRT1/Nrf2 pathway, suggesting enhanced antioxidant defense mechanisms. The scale bar is 100 μm. ** *p* < 0.01, and *** *p* < 0.001.

The SIRT1/Nrf2 signaling pathway plays a crucial role in the regulation of oxidative stress and cellular defense mechanisms, both of which are essential for effective wound healing. To investigate the potential involvement of this pathway in *C. militaris*-mediated wound healing, we performed immunofluorescence staining to assess the expression levels of SIRT1, Nrf2, and their downstream effector protein, HO-1 [40]. The immunofluorescence analysis demonstrated an obvious upregulation of both SIRT1 (Figure 9A) and Nrf2 (Figure 9B) expression in the *C. militaris*-treated group compared to the control group, suggesting that *C. militaris* actively modulates this critical signaling pathway. The increased expression of these proteins indicates enhanced activation of protective cellular pathways in response to *C. militaris* treatment. Furthermore, the analysis revealed a corresponding elevation in the expression of HO-1 (Figure 9C), a crucial antioxidant enzyme regulated by the SIRT1/Nrf2 pathway. HO-1 is widely recognized for its cytoprotective properties [40]. The upregulation of HO-1 in the *C. militaris*-treated group suggests that the activation of SIRT1 and Nrf2 leads to a downstream enhancement of antioxidant defense mechanisms, which may contribute to a more favorable microenvironment for wound healing. These findings collectively suggest that *C. militaris* not only activates SIRT1 and Nrf2 but also induces the downstream expression of HO-1, thereby strengthening the cellular defense against oxidative stress and facilitating the wound-healing process in normal and diabetic conditions.

## 4. Discussion

The present study provides strong evidence supporting the therapeutic potential of *Cordyceps militaris* in promoting wound healing, particularly in diabetic conditions where impaired healing is a major clinical challenge. Through a comprehensive evaluation using both in vitro and in vivo models, our findings demonstrate that *C. militaris* obviously accelerates wound closure, regulates key molecular pathways involved in tissue repair, and enhances skin barrier function, particularly in diabetic skin. Notably, *C. militaris* was found to modulate essential regulatory proteins associated with inflammation, oxidative stress, and cellular regeneration, contributing to an improved healing response. These results are consistent with previous research emphasizing the diverse pharmacological benefits of *C. militaris*, including its potent antioxidative, anti-inflammatory, and regenerative properties [15,16,17,18,19,20,21,22].

One of the key findings of this study is the capacity of *C. militaris* to upregulate HIF-1α and TGF-β1, both of which play essential roles in the complex processes of wound healing and tissue regeneration. HIF-1α is a key transcription factor that regulates cellular responses to hypoxia by promoting angiogenesis, enhancing oxygen supply, and facilitating metabolic adaptation in the wound microenvironment [30]. Accordingly, restoration or stabilization of HIF-1α has emerged as a promising therapeutic approach for enhancing chronic wound healing in diabetic patients by facilitating angiogenesis and promoting cell proliferation critical for tissue regeneration [41]. Similarly, TGF-β1 is a pivotal cytokine involved in various stages of wound repair, including the stimulation of fibroblast proliferation, differentiation, and extracellular matrix remodeling [38,42]. TGF-β1 plays a critical regulatory role in wound healing under diabetic conditions, and its inhibition has been shown to impair the wound repair process in DM. In this study, the observed increase in the expression levels of these factors in response to *C. militaris* supplementation suggests that this medicinal fungus may contribute to tissue repair. These findings highlight the potential of *C. militaris* as a promising bioactive agent in therapeutic strategies aimed at enhancing wound-healing outcomes.

Furthermore, the results of the immunofluorescence analysis demonstrated a significant upregulation of vimentin and PCNA in the *C. militaris*-treated group. These molecular markers play critical roles in various stages of wound healing and tissue repair. Vimentin, an intermediate filament protein, is a key regulator of epithelial-mesenchymal transition and fibroblast migration, both of which are essential for effective wound closure and tissue remodeling [43]. A deficiency in vimentin leads to impaired wound healing, manifesting as defects across multiple stages of the repair process. The presence of vimentin is essential for facilitating TGF-β signaling and driving the epithelial-to-mesenchymal transition, both of which are critical for effective re-epithelialization and tissue regeneration [43]. PCNA, a marker of cell proliferation, indicates enhanced cellular regeneration, while loricrin, a key structural protein in the epidermal barrier, suggests improved skin integrity [31,32,33]. MMP-1 and MMP-2 are essential regulators of wound healing through their roles in extracellular matrix remodeling. By degrading collagen and other matrix components, these enzymes enable the migration of keratinocytes, fibroblasts, and endothelial cells into the wound bed, thereby facilitating tissue repair and re-epithelialization [34,35,36]. In the present study, *C. militaris* treatment led to a notable elevation in the expression of MMP-1 and MMP-2. Considering that impaired matrix remodeling is a hallmark of diabetic wounds, the observed upregulation of these metalloproteinases indicates that *C. militaris* may help restore the dynamic extracellular matrix turnover required for efficient wound closure and tissue regeneration. These findings provide further support for the role of *C. militaris* in facilitating multiple stages of wound healing, from cellular migration and proliferation to epidermal differentiation.

In addition to promoting factors associated with wound repair, *C. militaris* treatment led to a notable decrease in IL-6 and TNF-α expression. IL-6 is a central mediator of the inflammatory response and is required for the normal progression of wound healing. Under physiological conditions, IL-6 expression decreases in the skin during the remodeling phase and returns to baseline levels; however, prolonged or excessive IL-6 expression can disrupt tissue repair and may be associated with delayed or impaired wound healing [37]. Similarly, TNF-α is a pro-inflammatory cytokine that plays a pivotal role in chronic inflammation and impaired wound healing, particularly in diabetic conditions [38,39]. The suppression of these inflammatory factors suggests that *C. militaris* exerts anti-inflammatory effects, which may contribute to a more favorable wound-healing environment by reducing excessive inflammation and promoting tissue regeneration.

The study further investigated the involvement of the SIRT1/Nrf2 signaling pathway in mediating the beneficial effects of *C. militaris* on diabetic wound healing and cellular protection. SIRT1 is an NAD+-dependent deacetylase that plays a crucial role in cellular homeostasis, particularly in modulating oxidative stress, inflammation, and metabolic regulation. Nrf2 is a key transcription factor responsible for orchestrating the cellular defense against oxidative damage by regulating the expression of antioxidant and cytoprotective genes [40]. Reduced Nrf2 activity exacerbates oxidative stress and inflammation, leading to delayed diabetic wound repair [44]. Activation of the SIRT1/Nrf2 signaling pathway has been demonstrated to promote diabetic foot ulcer healing [45]. Furthermore, the activation of the SIRT1/Nrf2 pathway led to a marked increase in the expression of HO-1, a downstream effector of Nrf2 that serves as a critical component of the antioxidant defense system [40,46]. Previous studies have confirmed that HO-1 activity alleviates oxidative stress and inflammatory responses, contributing to improved wound healing in diabetic conditions [47].

In this study, the significant upregulation of SIRT1 and Nrf2 observed in the *C. militaris*-treated group suggests that this medicinal fungus enhances the cellular response to oxidative stress, thereby promoting an environment conducive to tissue repair and regeneration. This effect is further supported by the upregulation of HO-1, a downstream effector of Nrf2, which indicates enhanced cellular ability against oxidative stress-induced damage—an essential factor in wound healing, where excessive reactive oxygen species impair fibroblast and keratinocyte function, ultimately delaying the repair process.

Moreover, even though murine models are extensively employed in dermatological research, the protective and therapeutic benefits of *C. militaris* aqueous extract demonstrated in the present study should be interpreted cautiously. This caution arises from well-recognized anatomical and physiological disparities between mouse and human skin, including hair follicle density, hydration status, differences in epidermal organization, biomechanical properties, subcutaneous composition, immune responses, and regenerative capacity [48,49,50,51]. Such interspecies variations may substantially affect the cutaneous response to topical interventions. Therefore, additional investigations using human-relevant experimental systems—such as ex vivo human skin models—are necessary to comprehensively assess the extract’s efficacy, safety profile, and dermal bioavailability. These efforts will be critical for enhancing translational relevance and supporting the systematic development of *C. militaris* aqueous extract as a potential therapeutic agent for wound healing in non-diabetic and diabetic conditions.

Despite the positive findings of this study, several limitations should be acknowledged. The aqueous extract of *C. militaris* is a complex mixture, and although adenosine and cordycepin were quantified, other potentially bioactive compounds were not systematically analyzed, making the specific contributions of individual compounds unclear. Mechanistic insights were mainly based on protein expression assessed by immunofluorescence, without direct evaluation of protein stability, downstream target gene regulation, oxidative stress markers, or pathway specificity using pharmacological inhibitors or genetic approaches. In addition, formal cytotoxicity, comprehensive in vivo safety assessments, and long-term wound outcomes such as tissue remodeling and scar maturation were not evaluated. While murine models provide valuable insight into diabetic wound healing, species-specific differences limit direct translation to humans. Future studies should incorporate bioactivity-guided fractionation, purified compound analyses, functional and quantitative pathway validation, standardized toxicity testing, extended observation periods, and clinically relevant formulations to better define the therapeutic potential of *C. militaris* in diabetic wound management.

Collectively, these findings underscore the potential of *C. militaris* as a promising natural therapeutic agent for enhancing wound healing. By accelerating wound closure, modulating key regulatory factors, exerting anti-inflammatory effects, and enhancing antioxidative defenses, *C. militaris* presents a multifaceted approach to wound repair. Further studies, including clinical trials, are warranted to validate these findings and explore the translational potential of *C. militaris* in diabetic wound management.

## 5. Conclusions

In conclusion, this study demonstrates that *C*. *militaris* exerts a multifaceted therapeutic effect on diabetic wound healing by modulating key biological processes involved in tissue repair. Treatment with *C. militaris* enhanced the expression of HIF-1α, while maintaining appropriate regulation of TGF-β1, a critical mediator of wound repair under diabetic conditions. In addition, the upregulation of MMP-1 and MMP-2 suggests improved extracellular matrix remodeling, thereby facilitating effective cell migration and re-epithelialization. *C. militaris* treatment also attenuated excessive inflammatory responses by reducing IL-6 and TNF-α expression, which are commonly associated with impaired healing in diabetic wounds. *C. militaris* also activated the SIRT1/Nrf2/HO-1 pathway, further indicating its role in strengthening antioxidative defenses, mitigating oxidative stress, and supporting wound healing. Collectively, these findings indicate that *C. militaris* promotes a balanced wound-healing microenvironment by coordinating matrix remodeling, oxidative stress decrease, and inflammation resolution. This integrated mode of action highlights the therapeutic potential of *C. militaris* as a promising natural intervention for the treatment of chronic diabetic wounds and warrants further investigation in translational and clinical settings.

## Figures and Tables

**Table 1:** Bioactive compounds of the *C. militaris* aqueous extract.

Compounds	Chemical	M.W.	Precursor Ion (*m*/*z*)	Sample (μg/mL)
Adenosine	C_10_H_13_N_5_O_4_	267.2	268.2	84.60
Cordycepin	C_10_H_13_N_5_O_3_	251.2	252.2	51.30

## Data Availability

The authors confirmed that the data supporting the findings of this study are available within the article. The raw data used and/or analyzed during the current study are available from the corresponding author upon request.

## Supplementary Materials

The following supporting information can be downloaded at: https://www.mdpi.com/article/10.3390/life16010117/s1. The report of HPLC/MS of the *C. militaris* aqueous extract.

## Abbreviations

The following abbreviations are used in this manuscript:

*C. militaris*	*Cordyceps militaris*
HPLC	High-performance liquid chromatography
MS	Mass spectrometry
TEWL	Trans-epidermal water loss
H&E	Hematoxylin and eosin
