# Correction: Lin et al. *Cordyceps militaris* Enhances Wound Repair Through Regulation of HIF-1α, TGF-β1, and SIRT1/Nrf2/HO-1 Signaling in Diabetic Skin. *Life* 2026, *16*, 117

**DOI:** 10.3390/life16091415

**Published:** 2026-08-26

**Authors:** Tzu-Kai Lin, Chia-Lun Tsai, Bruce Chi-Kang Tsai, Chia-Hua Kuo, Tsung-Jung Ho, Dennis Jine-Yuan Hsieh, Wei-Wen Kuo, Chih-Yang Huang, Pei-Ying Lee

**Affiliations:** 1 Department of Dermatology, Hualien Tzu Chi Hospital, Buddhist Tzu Chi Medical Foundation, Hualien 970473, Taiwan; 2 Department of Dermatology, School of Medicine, Tzu Chi University, Hualien 970374, Taiwan; 3 Cardiovascular and Mitochondrial Related Disease Research Center, Hualien Tzu Chi Hospital, Buddhist Tzu Chi Medical Foundation, Hualien 970473, Taiwan; 4 Laboratory of Exercise Biochemistry, The Education University of Hong Kong, New Territories, Hong Kong; 5 School of Physical Education and Sports Science, Soochow University, Suzhou 215006, China; 6 Department of Movement Sciences and Sports Training, School of Sport Sciences, University of Jordan, Amman 11942, Jordan; 7 Department of Chinese Medicine, Hualien Tzu Chi Hospital, Buddhist Tzu Chi Medical Foundation, Hualien 970473, Taiwan; 8 Integration Center of Traditional Chinese and Modern Medicine, Hualien Tzu Chi Hospital, Buddhist Tzu Chi Medical Foundation, Hualien 970473, Taiwan; 9 Department of Medical Laboratory and Biotechnology, Chung Shan Medical University, Taichung 402306, Taiwan; 10 Clinical Laboratory, Chung Shan Medical University Hospital, Taichung 402306, Taiwan; 11 Department of Biological Science and Technology, College of Life Sciences, China Medical University, Taichung 406040, Taiwan; 12 Ph.D. Program for Biotechnology Industry, China Medical University, Taichung 406040, Taiwan; 13 School of Pharmacy, China Medical University, Taichung 406040, Taiwan; 14 Graduate Institute of Biomedical Sciences, China Medical University, Taichung 404333, Taiwan; 15 Department of Medical Research, China Medical University Hospital, China Medical University, Taichung 404327, Taiwan; 16 Department of Medical Laboratory Science and Biotechnology, Asia University, Taichung 413305, Taiwan; 17 Division of Basic Medical Sciences, College of Nursing, Tzu Chi University, Hualien 970374, Taiwan

In the original publication [1], there was an error regarding the affiliation for “Tzu-Kai Lin”. In addition to “affiliation no. 1”, the updated affiliation should include: “Department of Dermatology, School of Medicine, Tzu Chi University, Hualien 970374, Taiwan”. The authors state that the scientific conclusions are unaffected. This correction was approved by the Academic Editor. The original publication has also been updated.
