# Supplementary material for: Cordyceps militaris Enhances Wound Repair Through Regulation of HIF-1α, TGF-β1, and SIRT1/Nrf2/HO-1 Signaling in Diabetic Skin
Source: Life (Basel). 2026 Jan 13;16(1):117. doi: 10.3390/life16010117 (PMC12843196; doi:10.3390/life16010117)
Supplement: Supplementary file 1 [file life-16-00117-s001.zip › life-4056742-supplementary.pdf]

Supplementary Material

HPLC/MS results

Instrument Device :

Instrument Categories	Instrument Model
Pump Model	Agilent 1260 G1312B Binary Pump
AutoSampler	Agilent 1260 G1367E HiP ALS Autosampler
Degasser	Agilent 1260 G1379B Degasser
Column	Phenomenex Kinetex-Phenyl-Hexyl-100A (100 mm x 2.1 mm i.d., 2.6 um)
Mass Spectrometer	AB Sciex Instruments QTRAP 5500
Source type	Turbo V Ion Source

HPLC Method Properties :

Duration : 10 min
Injection volume : 5 ul
Mobile Phase : A: 0.1% (v/v) Formic acid / Water ; B: 0.1% (v/v) Formic acid / Acetonitrile
Step Table :

Step	Total Time(min)	Flow Rate(μl/min)	A (%)	B (%)
0	0.10	100	85.0	15.0
1	2.00	100	85.0	15.0
2	5.00	100	70.0	30.0
3	6.00	100	10.0	90.0
4	7.00	100	10.0	90.0
5	8.00	100	85.0	15.0
6	10.0	100	85.0	15.0

Sample Preparation :

A volume of 100 μL of the sample was taken, followed by the addition of 200 μL of methanol. The mixture was vortexed thoroughly to ensure complete dissolution and subsequently incubated at -20°C for 30 minutes. Centrifugation was performed at 15,000 × g for 10 minutes, and the supernatant was collected and diluted 10× with 50% methanol prior to analysis. (Total dilution factor: 30×)

Mass Spectrometer Information :

Scan Type : MRM (Multiple Reaction Monitor)
Polarity : Positive ion mode
Source temperature : 400 °C
Data acquisition : Analyst 1.5 software

Parameter Table :

CUR (curtain gas) : 20.00 psi
Nebulizing gas (GS1) : 45.00 psi
Collision-activated Dissociation (CAD) : High
Heating gas (GS2) : 40.00 psi
Electrospray capillary voltage : 5500.00 V

Preparation of Calibrators :

The powdered standard was dissolved in methanol to prepare stock solutions of each target compound. These stock solutions were then used to prepare mixed working solutions with concentrations around µg/mL, which were stored at -20°C for future use. Calibration solutions were freshly prepared and serially diluted with 50% methanol to achieve a concentration range of ng/mL.

Quantitation Method :

As the provided standards are not isotope-labeled, the Multiple Point External Standard method was employed for quantitation.

Selected Transitions and Parameter Settings

Analytes	Q1 mass	Q3 mass	DP (V)	EP (V)	CE (V)	CXP (V)
Adenosine	268.2	85.2<br>136.2	180	10	34<br>24	13<br>10
Cordycepin	252.2	119.2<br>**136.2**	220	10	57<br>**21**	10<br>**12**

Note: Red indicates the ion pairs used for quantitation.

Calibration Curve of Standard

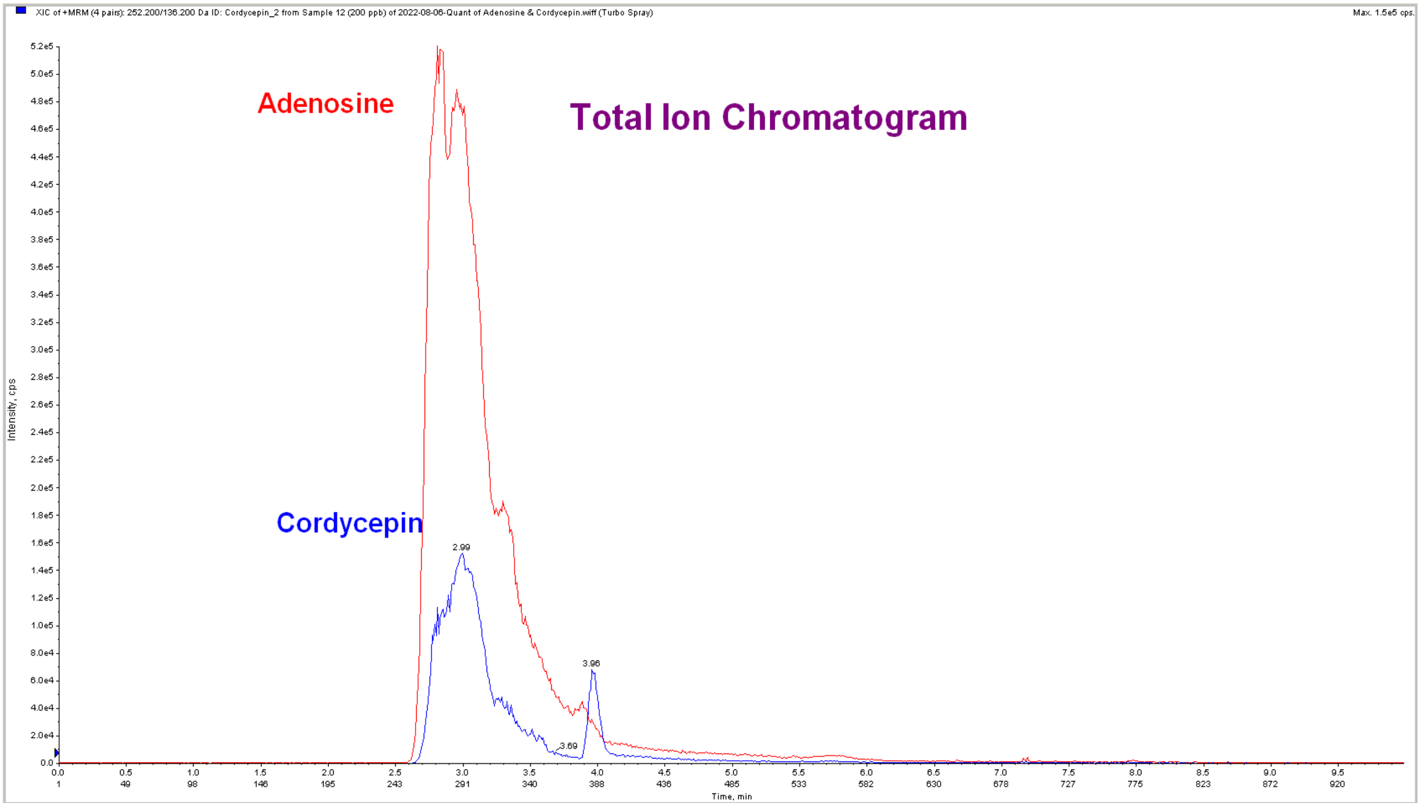

Note: Because Cordycepin is a derivative of Adenosine, the two have similar physicochemical properties and are difficult to completely separate under HPLC. However, the EIC chromatography results show that the selected ion pairs can all show significant

signals at their respective RT times, indicating their specificity.

Sample Spectrum

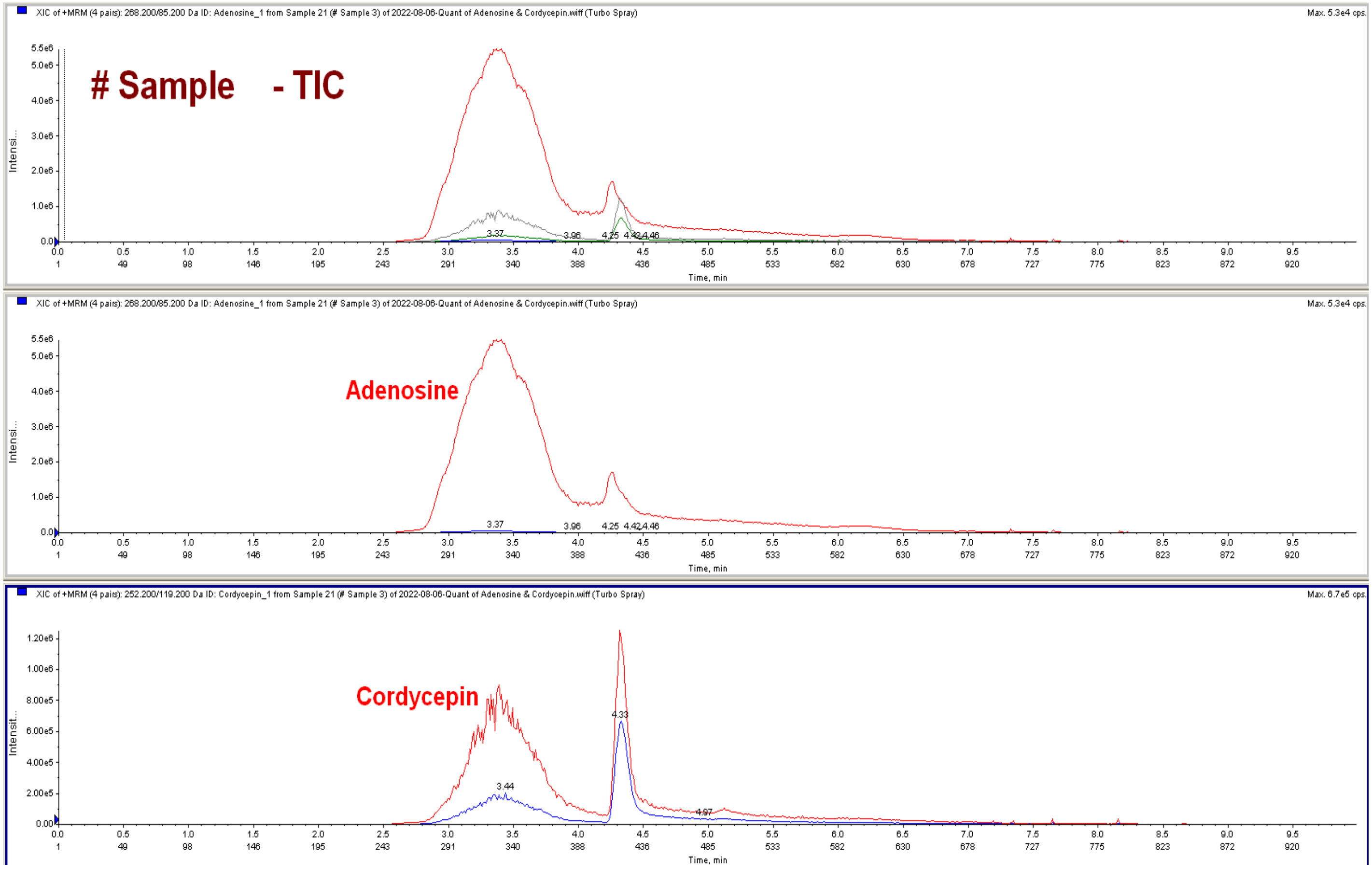

Analyte Name: Adenosine

Regression Equation: y = 6.56e+004 x + 3.87e+004 (r = 0.9999)

Expected Concentration (ng/ml)	Mean Calculated Concentration (ng/ml)	% Accuracy
0.32	0.33	104.3
0.64	0.62	97.2
1.6	1.64	102.6
3.2	3.24	101.4
8	7.90	98.8
16	15.67	97.9
40	38.64	96.6
80	79.88	99.9
200	203.47	101.7
400	398.35	99.6

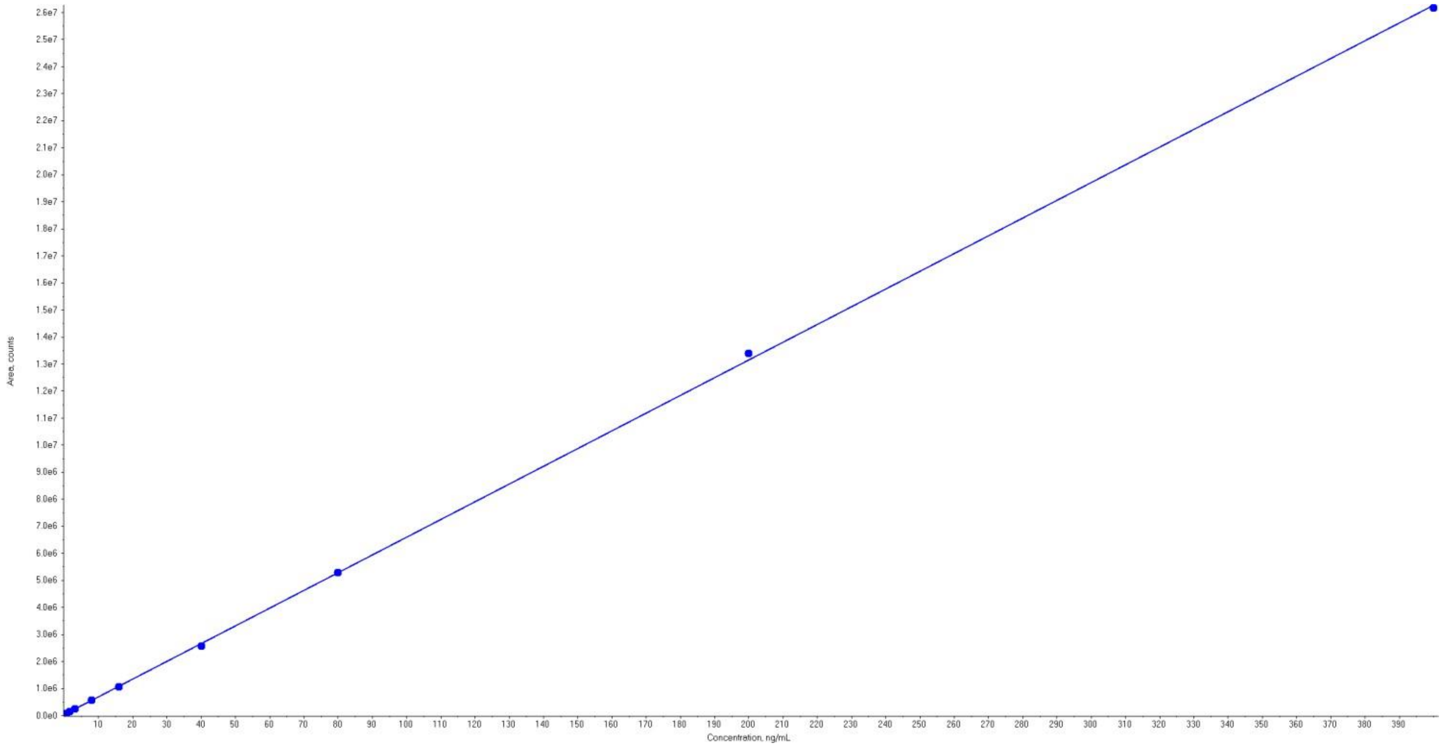

Analyte Name: Cordycepin
Regression Equation: y = 1.52e+004 x + 5.87e+004 (r = 0.9998)

**Expected Concentration (ng/ml)**	**Mean Calculated Concentration (ng/ml)**	**% Accuracy**
0.32	0.30	92.5
0.64	0.62	97.5
1.6	1.65	103.1
3.2	3.20	100.0
8	7.94	99.3
16	15.95	99.7
40	42.03	105.1
80	82.53	103.2
200	202.81	101.4
400	392.72	98.2

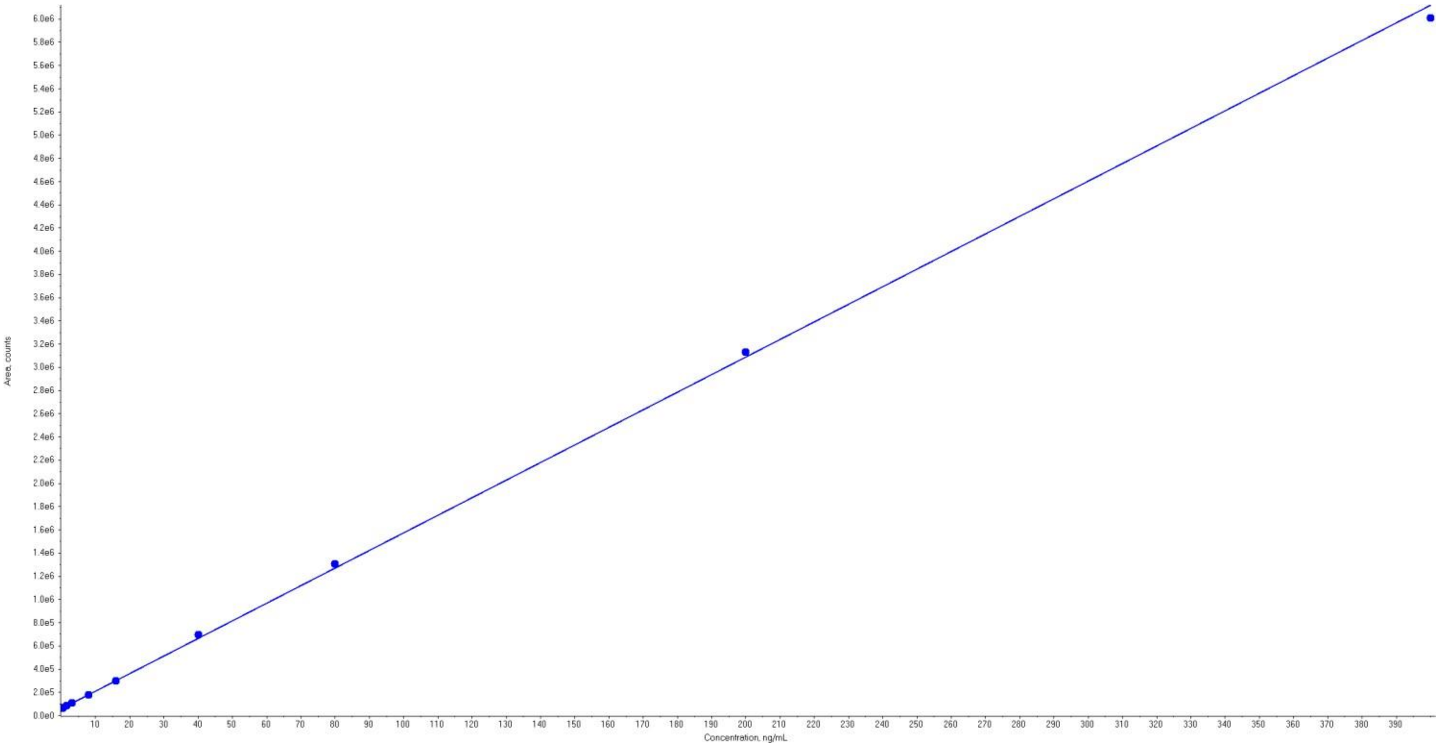

Results

Analyte Name	Spectrum	Calculated concentration (ng/ml)
Adenosine	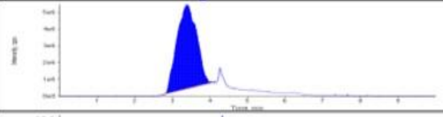	**2820.0**
Cordycepin	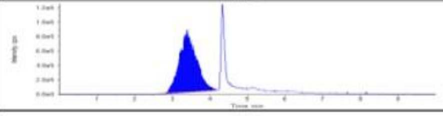	**1710.0**

Summary (The precise concentration of each metabolite in the sample; unit : ug/ml)
Calculation Formula: Analyte calculated concentration × 30 (dilution factor) / 1000

Analyte Name	Sample (μg/ml)
Adenosine	84.60
Cordycepin	51.30
